# Nutrition and Kidney Stone Disease

**DOI:** 10.3390/nu13061917

**Published:** 2021-06-03

**Authors:** Roswitha Siener

**Affiliations:** University Stone Center, Department of Urology, University Hospital Bonn, Venusberg-Campus 1, 53127 Bonn, Germany; Roswitha.Siener@ukbonn.de; Tel.: +49-228-287-19034

**Keywords:** calcium oxalate stone formation, diet, dietary assessment, fatty acids, fluid, oxalate, protein, sodium, uric acid, water

## Abstract

The prevalence of kidney stone disease is increasing worldwide. The recurrence rate of urinary stones is estimated to be up to 50%. Nephrolithiasis is associated with increased risk of chronic and end stage kidney disease. Diet composition is considered to play a crucial role in urinary stone formation. There is strong evidence that an inadequate fluid intake is the major dietary risk factor for urolithiasis. While the benefit of high fluid intake has been confirmed, the effect of different beverages, such as tap water, mineral water, fruit juices, soft drinks, tea and coffee, are debated. Other nutritional factors, including dietary protein, carbohydrates, oxalate, calcium and sodium chloride can also modulate the urinary risk profile and contribute to the risk of kidney stone formation. The assessment of nutritional risk factors is an essential component in the specific dietary therapy of kidney stone patients. An appropriate dietary intervention can contribute to the effective prevention of recurrent stones and reduce the burden of invasive surgical procedures for the treatment of urinary stone disease. This narrative review has intended to provide a comprehensive and updated overview on the role of nutrition and diet in kidney stone disease.

## 1. Introduction

The prevalence of urolithiasis of the general population has increased worldwide in the past decades and was reported to be 4.7% in Germany and up to 10.1% in the United States [1,2,3]. The recurrence rate of urinary stones is high and is estimated to be approximately 50% at 10 years [4]. Nephrolithiasis is associated with an increased risk of chronic and end-stage kidney disease, probably due to kidney injury from obstructive nephropathy [5,6]. The most common stone type is calcium oxalate (67%) followed by calcium phosphate (17%), uric acid (8%), struvite (3%), and cystine (0.4%) [7]. Urinary stone formation is a multifactorial process to which metabolic derangements, genetic factors, anatomical and functional abnormalities may contribute, whereby nutrition plays a crucial role. Diet composition can affect urine risk profile and the supersaturation with the stone-forming salt, which can modify the risk of urinary stone formation [8,9].

A reliable stone analysis is an essential precondition for specific treatment regimens, because urinary risk factors for stone formation vary with the type of stone [10,11]. Moreover, a thorough metabolic evaluation of the stone patient is required, including detailed medical history, dietary assessment as well as blood and urine analysis [11,12,13,14,15,16,17]. To reduce the risk of recurrent stone formation, targeted dietary treatment should be individually adjusted to the metabolic risk profile of each patient. The collection of two consecutive 24 h urines is recommended to detect frequent metabolic disturbances such as hypercalciuria, hypocitraturia, hyperoxaluria, and hyperuricosuria and to identify dietary risk factors for kidney stone formation. Specific nutritional therapy, based on dietary assessment and metabolic evaluation, has been demonstrated to be more effective than general dietary measures in preventing recurrent stone formation [18]. The aim of this narrative review is to summarize the current knowledge of the role of nutrition in kidney stone formation.

## 2. Fluid Intake

A low urine volume caused by insufficient fluid consumption or excessive fluid loss is one of the most crucial risk factors for kidney stone formation [19]. Numerous physiological and pathological conditions may lead to dehydration that modifies water needs. These factors include excessive sweating due to heat exposure, mental stress, high physical activity level, and occupation as well as chronic diarrhea in the setting of fat malabsorption due to different gastrointestinal disorders [19,20,21]. A study of 100 steel plant workers reported a history of stone disease in 16%, with a urine osmolality of more than 700 mOsm, defined as dehydration, in more than 50% of workers [22]. Occupations at elevated risk for stone formation also include health care professionals with limited access to water. A survey of employees found that physicians working in the operating room had the highest prevalence of nephrolithiasis (17.4% vs. 9.7%) and reported higher stress levels and lower fluid intake compared to those who work at other locations [23]. Poor access to fluids or to bathroom facilities are other circumstances, in which occupation can affect urolithiasis, such as, for example, in professional drivers, airplane pilots or schoolteachers [24].

A randomized controlled study of 199 first-time idiopathic calcium oxalate stone patients evaluated the impact of a high fluid intake in preventing stone recurrence [25]. Patients were randomly assigned to a high water intake to achieve a urine volume of at least 2 L/day or to a control group without specific instructions. After the five-year follow-up period, the intervention group was found to have a 2.5-fold higher urine volume, a lower recurrence rate (12% vs. 27%; *p* = 0.008) and a longer time to recurrence (38.7 months vs. 25.1 months; *p* = 0.016) than the control group. Several systematic reviews and meta-analyses on the role of fluid intake in the secondary prevention of urolithiasis concluded that high total fluid intake to achieve a urine volume of greater than 2.0 to 2.5 L/day decreases the risk of stone recurrence [26,27,28,29,30,31]. While fluid intake was reported to be a protective factor in the primary prevention of kidney stone formation in cohort studies [30], no evidence was found from randomized controlled trials [31].

An adequate fluid intake is the most important nutritional measure to prevent kidney stone recurrence, regardless of urine stone composition and individual risk factors for stone formation [19]. The intake of an adequate amount of fluid increases urine dilution, thereby reducing the concentration of lithogenic constituents and encouraging the expulsion of crystals by decreasing the renal intratubular transit time [32]. According to the guidelines on urolithiasis, a copious fluid intake to maintain a urine volume of at least 2.0 to 2.5 L/24 h is recommended for most types of stones [15,16]. For the recurrence prevention of patients with cystinuria, excessive urine dilution is of the utmost importance. In adult cystine stone patients, urine volume of at least 3.0 L/24 h should be achieved to decrease urinary cystine concentration to below the solubility limit of 1.3 mmol/L at pH 6.0 [14,15,33]. A study of 27 adult patients with cystinuria demonstrated that maintaining a urine volume of more than 3.0 L/day significantly reduced recurrent stone formation [34]. The specific recommendations regarding fluid intake are summarized in Table 1.

While the benefit of high fluid intake has been confirmed, the effect of different beverages is still debated. The constituents of different beverages can affect urine composition and consequently the risk of stone formation.

### 2.1. Tap Water and Mineral Water

The impact of the composition of tap water and mineral water on kidney stone formation is still a matter of debate. The composition of drinking water, predominantly the content of the divalent cations calcium and magnesium, varies widely between geographic regions within the same country [35]. Hard tap water, defined as a calcium carbonate concentration above 120 mg/L [36], can contribute to the daily dietary calcium intake. A study on 2295 patients from two regions in the United States found an increased risk of urinary stone formation for individuals consuming tap water from a private well [37]. However, the cause of elevated risk by private well users is unknown. Other cohort studies have not identified an association between the hardness of water from public water suppliers and urinary stone disease [37,38,39].

In contrast to tap water, bicarbonate is a natural constituent of mineral water besides calcium, magnesium and other ions. The intake of bicarbonate increases the buffering capacity of the body and is a strong alkalizing agent. Mineral water rich in bicarbonate can support alkalinization therapy and contribute to inhibitory potential of urine by increasing urine pH and citrate excretion [40]. A randomized crossover trial of healthy subjects compared the effect of an equimolar alkali load in form of bicarbonate-rich mineral water or potassium citrate on the urinary risk profile for calcium oxalate and uric acid stone formation [41]. The intake of 2 L/day of mineral water containing 1715 mg/L bicarbonate or 2.55 g/day potassium citrate significantly increased urine pH and citrate excretion and decreased oxalate excretion. The relative supersaturation of calcium oxalate and uric acid declined significantly in both groups. A study of healthy individuals conducted under controlled dietary conditions demonstrated that a mineral water rich in bicarbonate, calcium and magnesium increased urine pH, as well as the excretion of the urinary inhibitors of calcium oxalate stone formation citrate and magnesium [42]. Despite the significant increase in calcium excretion, no change in the relative supersaturation of calcium oxalate occurred.

A study of 22 idiopathic calcium oxalate stone patients evaluated the impact of three mineral waters with different contents of bicarbonate and calcium. The intake of the mineral water rich in bicarbonate and calcium resulted in a significant increase in urinary citrate and decrease in urinary oxalate excretion, while urinary calcium excretion and the supersaturation of calcium oxalate did not change [43]. A study by Rodgers [44] found that the intake of 1.5 L/day of a mineral water with high calcium content (202 mg/L) compared to 1.5 L/day of a tap water with low calcium content (13 mg/L) significantly increased calcium excretion in 20 healthy individuals of each sex as well as 20 female calcium oxalate stone formers but not in male patients. Urinary oxalate excretion and the relative supersaturation of calcium oxalate did not change in any of the four groups. A double-blind crossover study of 34 recurrent calcium oxalate stone patients under a regular diet examined the effect of 1.5 L/day of a bicarbonate-rich mineral water (2673 mg/L) vs. 1.5 L/day of a mineral water with a low bicarbonate content (98 mg/L) on the risk of urinary stone formation [45]. The intake of the bicarbonate-rich mineral water resulted in a significant rise in urine pH, citrate and magnesium excretion compared to the control. The relative supersaturation of calcium oxalate decreased to a similar extent in both groups.

The impact of mineral water on the risk of calcium oxalate and uric acid stone formation is mainly determined by the presence of bicarbonate [40,41,45]. The effect of bicarbonate-rich mineral water corresponds to that of sodium bicarbonate in galenic form [46]. If calcium in high concentration is present in the water, the positive effect of increased urine pH and citrate excretion is neutralized by an increased urinary calcium excretion [42,44]. A randomized trial of 129 healthy women and men found that the consumption of at least 1.5 to 2.0 L/d of mineral water rich in bicarbonate (>1800 mg/L) can decrease the net acid excretion by reducing the dietary acid load [47]. Urine alkalinization is an important nutritional measure in the treatment of patients with calcium oxalate, uric acid and cystine stones, but it is not indicated in calcium phosphate and struvite stone disease [14]. Comparisons of the composition of commercial bottled ‘still’, ‘carbonated’ and ‘sparkling’ water from 10 European countries found a wide variation in the calcium content, with concentrations reaching up to 581.6 mg/L [48,49]. As the dietary reference intake of calcium of a total of 1000 to 1200 mg per day are already achieved by consuming 2 L of calcium-rich water, patients should be aware of the calcium content of the water.

### 2.2. Fruit Juices and Fruit Juice Beverages

The metabolic effect of fruit juices is primarily determined by their alkali citrate content. Dietary citrate is absorbed in the gastrointestinal tract and metabolized to bicarbonate, which may then increase urine pH and citrate excretion [50]. Citrus juices, such as lemon, orange and grapefruit juice, supply large quantities of citric acid and could be a dietary alternative to the pharmacotherapy with alkalizing agents. Orange juice is among the most popular fruit juices consumed worldwide. Findings from studies on the impact of orange juice intake on urinary risk factors for stone formation are mixed.

Three cohort studies reported that the intake of orange juice is associated with a reduced risk of kidney stone formation [51]. Interventional studies under a controlled dietary regimen showed that orange juice provided an alkali load that increased urine pH and citrate excretion [52,53,54]. Despite the alkalizing effect, orange juice did not change the calculated risk for calcium oxalate in the majority of studies. Although the oxalate concentration of orange juice is very low (Table 2) [55,56], two of the three studies reported a significant rise in urinary oxalate excretion, which could be due to in vivo conversion of ascorbate to oxalate [52,54]. Due to concerns over the high sugar and energy content and the lack of dietary fiber of orange juice, the nutritional advice is to opt for the whole fruit over the juice, to limit daily consumption of fruit juice to one serving and to dilute the juice with water [14]. A randomized crossover study of 10 healthy subjects under regular diet comparing a crystal light lemonade beverage and two low-calorie orange juice beverages noted a higher urine pH as the only significant change between the groups when participants consumed Kroger low-calorie orange juice beverage [57]. However, citrate excretion, a major urinary inhibitor of calcium oxalate stone formation, did not significantly differ between the groups. Moreover, concerns over health risks associated especially with overconsumption and with certain artificial colorings, preservatives, sweeteners and additives, such as ascorbic acid and calcium, severely limit the health benefit of these beverages for kidney stone formers [57,58].

Compared to orange juice, lemon and lime juices were found to have higher citrate concentrations [61]. However, findings from studies on the impact of lemon juice and lemonade on urinary risk factors for stone formation are likewise inconclusive. Although several studies in stone patients with or without hypocitraturia found a significant rise in urinary citrate excretion after the administration of lemonade [62,63,64,65], others failed to confirm the citraturic effects of regular lemonade [54,66,67]. Reasons for the variability in urinary citrate excretion with lemonade could be different degrees of dilution of pure lemon juice and the higher proportion of non-alkaline citric acid, which could have neutralized the alkali potential of citrate [57,68].

Studies of other fruit juices, such as grapefruit, apple, cranberry, and blackcurrant juice, have also provided inconsistent results. The findings of two studies of healthy subjects on the impact of grapefruit juice yielded no or only a partial effect on the risk of calcium oxalate stone formation (500 mL/day: *p* < 0.05; 720 mL/day: n.s.; 1000 mL/day: n.s.), although urinary citrate excretion increased significantly [53,69]. The administration of 0.5 or 1.0 L/day of apple juice likewise increased urinary citrate excretion but did not change the relative supersaturation of calcium oxalate [53]. In healthy volunteers, the consumption of 330 mL/day of blackcurrant juice significantly increased urine pH and citrate excretion [70]. However, a simultaneous rise of urinary oxalate excretion was observed, probably due to in vivo conversion of ascorbate to oxalate. Interventional studies on the effect of cranberry juice reported that oxalate excretion decreased [71], remained unchanged [72] or increased [70] in healthy subjects and increased in calcium oxalate stone patients [72]. Overall, the relative risk of calcium oxalate stone formation was unaffected [70,72] or reduced [71] in healthy volunteers but increased in stone patients after the intake of cranberry juice [72]. Finally, a study of healthy subjects showed that the consumption of 1.9 L/day of coconut water compared to tap water significantly raised urinary citrate, potassium, and chloride excretion, without affecting urine pH [73]. Although fruit and vegetable juices could be useful in the dietary therapy of kidney stone disease, the oxalate concentration has to be taken into account.

### 2.3. Soft Drinks

One large randomized controlled trial in stone patients with a soft drink intake of at least 160 mL/day randomly assigned men to refrain from soft drink consumption or to the control group [74]. The study showed that the consumption of soft drinks, especially those acidified by phosphoric acid, significantly increased the risk for recurrent stone formation. The analysis of data from 194,095 participants in the Health Professionals Follow-Up Study (HPFS), and Nurses’ Health Study (NHS) I and II with a median follow-up of more than eight years indicated significant positive associations with the risk of stone formation for sugar-sweetened cola and sugar-sweetened non-cola [51]. According to findings from a cross-sectional trial based on the Third National Health and Nutrition Examination Survey (NHANES-III), sugar-sweetened soft drink consumption was positively associated with serum uric acid concentration and frequency of hyperuricemia [75]. Accordingly, cohort studies also indicated a strong positive association between sugar-sweetened soft drinks and the risk of gout in men [76]. These results could at least partly be explained by the fructose content of sugar-sweetened soft drinks, which has been associated with an increased risk of incident kidney stone formation [30,77].

### 2.4. Tea and Coffee

Tea and coffee are among the most commonly consumed beverages. A systematic review and large cohort studies supported in general a potentially preventive role for both coffee and tea consumption against stone formation [30,51,78,79,80]. It is assumed that the beneficial effect of tea and coffee could be attributed primarily to the diuretic action of the consumption of large amounts of caffeine, which could offset, at least partially, the hypercalciuric effect [78,81,82]. The European Food Safety Authority considers the habitual caffeine consumption of up to 400 mg/day, corresponding to about four cups of brewed coffee, a safe amount for healthy adults, except pregnant women [83]. An increased total fluid intake and the antioxidative effect of phytochemicals, such as polyphenols, could be other explanations for the preventive effect of tea consumption [78,84].

A limitation of the cohort studies is that no distinction was made between different coffee and tea types, such as black, green and herbal tea. Moreover, there has been concern regarding the oxalate content of coffee and tea. While the oxalate content of coffee is low [55], black tea and green tea contain varying amounts of oxalate depending on the origin, quality, time of harvest and preparation [59,60,85] (Table 2). The highest oxalate concentration was detected in black and green tea [59,60,86], whereas other types of tea such as herbal and fruit tea were found to be low in oxalate [60,86]. Therefore, the exact mechanism for the protective effect of black and green tea against stone formation remains to be elucidated.

## 3. Protein

The recommended intake of protein for adults is between 0.8 and 1.0 g per kg normal body weight per day [87]. High dietary intake of protein was reported to exert potential detrimental effects on urinary risk factors of stone formation. The acid load provided by a high protein intake may increase urinary calcium and reduce urine pH and citrate excretion [88,89]. A study of 18 hypercalciuric stone patients showed that a protein restriction to 0.8 g/kg body weight/day decreased urinary calcium and uric acid and increased urinary citrate excretion [90].

However, the evidence from systematic reviews regarding the relationship between protein intake and the risk of kidney stone formation is inconsistent [91,92]. The two cohort studies [93,94] included in the systematic review by Pedersen [91] found no association between dietary protein intake and stone formation. However, one systematic review confirmed that high-protein diets were associated with increased urinary calcium excretion, which is a risk factor for calcium stone formation [95]. In healthy subjects, the administration of 1.5 g per day L-methionine did not raise urinary calcium excretion [96], whereas the intake of 3 g per day L-methionine significantly increased calcium excretion by about 1 mmol per day [97]. To date, there is no randomized controlled trial comparing the isolated effect of a high versus low protein intake on the risk of urinary stone formation.

While findings on the association between dietary protein consumption and the risk of stone formation are inconclusive, large observational studies found that a higher dietary net acid load was associated with higher risk of stone formation [98]. These data suggested that the proportion of consumed vegetables and fruits compared to ingested protein intake, rather than total protein per se, could be a more reliable indicator for the risk of urinary stone formation. Vegetables and fruits have a distinct alkalizing potential and can neutralize the proton load, metabolically generated from ingested protein [99,100]. In hypocitraturic stone patients, the introduction of vegetables and fruits increased urine pH and citrate excretion and reduced the relative supersaturation of calcium oxalate and uric acid [101]. Reduced urine pH and citrate excretion, resulting from high nutritional proton load or high dietary acidity, are risk factors for several types of urinary stones, particularly for the most common, i.e., calcium oxalate and uric acid. The higher the urine pH, the higher the stone-inhibiting citrate excretion and calcium-binding capacity and the lower the urinary calcium excretion [102].

## 4. Carbohydrates

Findings from studies on the impact of carbohydrates on the risk of kidney stone formation are inconclusive. While some studies reported a similar intake of carbohydrates in stone patients and controls [103,104], others noted a higher intake of carbohydrates in stone patients than in controls [105,106]. A major limitation of these trials is that they did not distinguish between different types of carbohydrates, especially the most common disaccharide sucrose and its monomers glucose and fructose.

Prospective cohort studies found a positive relationship between sucrose consumption and the risk for stone formation in women but not in men [93,94,107,108]. A previous study reported a higher rate of urinary calcium excretion after oral ingestion of 100 g glucose or sucrose in normal subjects and calcium oxalate stone formers, where the response was even more pronounced in the latter group [109]. The rise in calcium excretion after an oral glucose load has been attributed to an increased intestinal absorption and reduced renal tubular reabsorption of calcium [110,111,112]. It was suggested that this effect could be, at least partially, mediated by an increase in serum insulin. A study of calcium stone patients with idiopathic hypercalciuria and healthy controls conducted using a fixed metabolic diet concluded that hyperinsulinemia is unlikely to play a significant role in the pathogenesis of calcium stone formation among patients with idiopathic hypercalciuria [113].

Consumption of fructose has clearly increased over the last decades as fructose is used as sweetener in beverages or food as a replacement for sucrose or glucose. A systematic review and meta-analysis noted a positive relationship between fructose intake and the risk of incident stone formation [30], but the underlying mechanisms are not well-understood [114]. Fructose consumption was assumed to increase the risk of stone formation in part via effects on the urinary excretion of calcium [115], oxalate [115,116], urine pH [116], and by effects on uric acid metabolism [115,116,117,118]. A cohort study of men showed a positive association between fructose intake and the risk of incident gout [76]. Studies on fixed metabolic diets are required to evaluate the effect of sucrose, glucose and fructose on metabolism and urinary risk factors for uric acid and calcium oxalate stone formation.

## 5. Fat

Data regarding the association between dietary fat consumption and the risk of urinary stone formation are scarce and inconsistent. While several studies reported a similar fat intake in stone patients and controls [103,104], others found higher dietary fat intake in stone formers [105,106].

Studies have suggested that the dietary fatty acid pattern, especially the ratio of n-6 to n-3 polyunsaturated fatty acids, may affect the risk of calcium oxalate stone formation through various complex mechanisms [119]. An abnormal concentration of arachidonic acid (C20:4n-6) in plasma and erythrocyte membrane phospholipids was found in idiopathic calcium oxalate stone patients compared to healthy controls [120]. Arachidonic acid in cell membrane phospholipids can be released by phospholipase enzymes and subsequently serve as a precursor of PGE_2_ [121,122]. An elevated PGE_2_ production is assumed to induce hypercalciuria by increasing intestinal calcium absorption and bone resorption [123,124], and by decreasing renal tubular calcium reabsorption [125,126]. Increased phospholipid arachidonic acid levels may also induce hyperoxaluria by activating anion carriers and consequently the intestinal and renal transport activity of oxalate [120,127]. A study of 20 healthy volunteers reported that the supplementation of n-3 polyunsaturated fatty acids DHA (22:6n-3) and EPA (20:5n-3) led to their incorporation into cell membrane phospholipids, which was partly at the expense of arachidonic acid [128]. A change in the membrane fatty acid pattern due to increasing dietary intake of n-3 polyunsaturated fatty acids, therefore, was assumed to decrease the urinary excretion of calcium and oxalate.

Analyses of 24 h urine samples and dietary records of 58 idiopathic calcium oxalate stone patients showed a positive association between the dietary arachidonic acid content and urinary oxalate excretion [129]. Several studies investigated the role of fish oil administration in the dietary management of stone formation. Fish oil supplementation was found to reduce oxalate excretion in healthy subjects [130], and to decrease urinary excretion of calcium and/or oxalate in calcium stone patients in most trials [119]. Further studies should identify those patients who benefit most from n-3 polyunsaturated fatty acid supplementation.

## 6. Oxalate

Urinary oxalate is regarded as an essential risk factor for calcium oxalate stone formation. Changes in urinary oxalate concentration can significantly increase the urinary supersaturation of calcium oxalate [131,132]. A prospective study of 134 recurrent calcium oxalate stone patients identified the rise in oxalate excretion as the major urinary determinant for relapse after a two-year follow-up [133]. Oxalate is an end product of metabolism. Urinary oxalate is derived from endogenous oxalate synthesis and dietary oxalate intake [134]. Endogenous oxalate metabolism occurs predominantly in the liver and is affected by dietary intake of precursors, such as ascorbic acid and hydroxyproline [135,136].

The effect of dietary oxalate intake on urinary oxalate excretion and the risk of stone formation has been examined in several interventional trials. In a study of healthy subjects, the mean contribution of dietary oxalate to urinary oxalate excretion ranged from 24% (10 mg/day dietary oxalate) to 42% (250 mg/day dietary oxalate) [137]. A study of 20 healthy women and men showed that a controlled oxalate-rich diet (600 mg/day dietary oxalate) compared to a diet normal in oxalate (100 mg/day dietary oxalate) significantly increased oxalate excretion from 0.354 to 0.542 mmol/24 h by 0.188 mmol/24 h, i.e., >50%, corresponding to 35% of total urinary oxalate excretion [138]. This study also showed that the supersaturation of calcium oxalate increases significantly with a high dietary oxalate intake. However, a prospective cohort study reported only a modest positive association between dietary oxalate intake and the risk for incident stone formation [139]. Several reasons could be responsible for these inconsistencies, including the use of food frequency questionnaires, which are prone to errors, to evaluate dietary oxalate intake in large cohort studies, the daily variation in the oxalate ingestion and the variability of the oxalate content due to growth conditions, preparation and processing of food [59,140,141,142]. Therefore, studies using diets strictly controlled in their oxalate and nutrient content and the use of comprehensive data on the oxalate content of raw and processed foods are required.

Dietary oxalate is mainly derived from plant foods. Estimates of dietary oxalate intake are in a wide range, depending on the ingestion of oxalate-rich foods. Therefore, it is essential to consider sources of excess dietary oxalate. Detailed knowledge of the oxalate content of foods is important in the dietary therapy of calcium oxalate stone patients. However, the previous lack of accurate and complete data on the oxalate concentration of foods hindered the elucidation of the role of dietary oxalate in urinary oxalate excretion and the risk of stone formation. Analysis of the oxalate content of a wide variety of foods by a HPLC enzyme-reactor method provided a comprehensive database of the oxalate content of foods and beverages and detected a considerable number of foods with high or extremely high oxalate concentrations [55,56,59,60,143,144,145]. An overview of foods rich in oxalate, including vegetables, legumes, fruits, cereals and pseudocereals, nuts, herbs and spices, is presented in Table 3.

As boiling may lead to considerable losses of oxalate into the cooking water, food processing and preparation methods are important determinants for the oxalate content [141]. For example, the oxalate concentration of raw spinach was found to be more than five times higher in raw (1959 mg/100 g) compared to cooked spinach (364 mg/100 g) [55,143]. To avoid losses of water-soluble vitamins and minerals, it is recommended to shortly steam spinach, which, however, also preserves most of the oxalate content of raw spinach. Moreover, beverages such as vegetable and fruit juices as well as black, green and iced teas were found to contain considerable amounts of highly bioavailable oxalate (Table 2) [56,59,60]. Knowledge of the oxalate concentration of beverages is particularly crucial as a high fluid intake is the most important nutritional measure for recurrence prevention of kidney stone disease. Kidney stone formers should be advised to avoid oxalate-rich foods and beverages.

A case-control study of 186 calcium oxalate stone patients found a significant positive association between dietary ascorbic acid intake and urinary oxalate excretion [134]. The association between ascorbic acid intake and the risk of urinary stone formation has been noted in several large cohort studies [146,147]. A study under controlled dietary conditions noted a significant increase in urinary oxalate in both calcium oxalate stone patients and healthy controls after oral supplementation of 2 g ascorbic acid daily [148]. Dietary hydroxyproline, mainly present in collagen/gelatin, may also contribute to endogenous oxalate synthesis and urinary oxalate excretion in healthy subjects and in primary hyperoxaluria [135,149,150]. A study using infusions of [^15^N,^13^C_5_]-hydroxyproline found that hydroxyproline contributed at least 15% to urinary oxalate excretion in healthy volunteers and could be a major source of the oxalate produced in patients with primary hyperoxaluria [149,150].

Intestinal hyperabsorption of oxalate may additionally contribute to urinary oxalate, even in the absence of intestinal diseases associated with fat malabsorption. A study under standardized conditions using [^13^C_2_]oxalate demonstrated that the mean intestinal oxalate absorption was significantly higher in 120 idiopathic calcium oxalate stone patients compared to 120 healthy subjects (10.2% vs. 8.0%; *p* < 0.001) [151]. Intestinal hyperabsorption, defined as oxalate absorption exceeding 10%, was noted in 28% of healthy volunteers and in 46% of patients. The amount of dietary oxalate that is intestinally absorbed is affected by dietary constituents, especially the calcium intake [152]. Moreover, enteric hyperoxaluria in the setting of fat malabsorption due to different gastrointestinal disorders is a crucial risk factor contributing to kidney stone formation [153,154,155]. Unabsorbed fatty acids bind to calcium, decreasing the intraluminal calcium concentration for complexation with oxalate [156]. With the depletion of free calcium, a larger percentage of unbound oxalate is available for absorption in the gut. A study of 51 patients with intestinal fat malabsorption found that the resection status is a major risk factor for hyperoxaluria and kidney stone formation [157].

Finally, the intestinal colonization with the Gram-negative anaerobic oxalate-degrading bacterium *Oxalobacter formigenes* can be inversely associated with calcium oxalate stone formation [158]. Although the administration of *Oxalobacter formigenes* or other probiotic preparations was suggested to reduce urinary oxalate excretion and the lithogenic risk, findings from several interventional studies are conflicting [138,159]. Future studies should include functional and nutritional aspects of the interaction between nutrients and the gut microbiota composition.

## 7. Calcium

Hypercalciuria is a crucial risk factor for calcium stone formation. Balanced dietary calcium intake from both dairy and non-dairy sources has been demonstrated to exert a preventive effect against urinary stone formation [160]. Dietary calcium restriction should be avoided as it may induce bone loss and result in the hyperabsorption and hyperexcretion of oxalate. Dietary calcium restriction reduces intestinal calcium concentration, which enhances the absorption of uncomplexed oxalate and subsequently urinary oxalate excretion [152]. A study of healthy volunteers with a standardized [^13^C_2_]oxalate absorption test showed that intestinal oxalate absorption was inversely associated with dietary calcium intake within the range of 200 (17% oxalate absorption) to 1200 mg/day calcium (2.6% oxalate absorption) [152]. Dietary calcium intake above 1200 mg per day had only a minor impact on oxalate absorption. Epidemiologic studies found an inverse relationship between dietary calcium intake and the risk of stone formation in women and men [93,107,108]. A five-year prospective randomized study of 120 men with calcium oxalate stone formation and hypercalciuria showed that recurrences were less frequent on a normal calcium (1200 mg/day), normal-protein, and low-salt diet compared to a low-calcium diet (400 mg/day) [161]. For idiopathic calcium stone formers, a total dietary calcium intake of 1000 to 1200 mg/day is recommended [14,15,16].

## 8. Sodium Chloride

Dietary sodium chloride consumption increases the risk of stone formation due to its propensity to enhance urinary calcium excretion [162]. High sodium chloride intake may promote calcium excretion by inhibiting renal tubular calcium reabsorption from the sodium-induced expansion of extracellular fluid volume [163]. Interventional studies in normal adults showed that every 100 mmol (2300 mg) increase in sodium intake per day enhances daily calcium excretion by approximately 1 mmol [164]. A correlation between dietary sodium intake and kidney stone formation was observed in one cohort study [108], but was not confirmed by others [93,94,107]. These discrepancies may reflect the difficulties in obtaining reliable estimates of dietary salt intake based on food frequency questionnaires. A randomized controlled trial of 210 idiopathic calcium oxalate stone patients demonstrated that a low-salt diet could effectively decrease urinary calcium excretion compared to a control diet [165]. The recommended dietary sodium intake is <100 mmol (2300 mg) or 6 g of salt (sodium chloride) per day [14,16,17].

## 9. Dietary Management

Diet modification is an effective method to correct urinary risk factors for kidney stone formation, particularly of the most common stone type calcium oxalate. Dietary therapy should be tailored to every single patient according to the patient-specific biochemical and dietary risk profile. A detailed nutritional assessment is an essential component of the evaluation and the main prerequisite for a successful dietary therapy of the stone-forming patient. Seven-day dietary records are regarded as the most accurate technique for assessing habitual dietary intake. A variety of dietary factors, including fluid intake, dietary protein, carbohydrates, oxalate, calcium, and sodium chloride can modulate urinary risk profile and contribute to the risk of kidney stone formation. The specific dietary recommendations for calcium oxalate stone formers are included in Table 4.

## 10. Conclusions

Nutritional factors play an important role in kidney stone formation. A careful dietary assessment should obtain from the patient any dietary habits that predispose them to urinary stone formation. An appropriate diet can modulate the urinary risk profile and contribute to the reduction of the risk of urinary stone formation. Specific dietary therapy, based on nutritional assessment and metabolic evaluation, has been demonstrated to be more effective than general dietary measures in preventing recurrent stone formation.

## Figures and Tables

**Table 1:** Recommendations for fluid intake—adapted from [14,15,16].

**General Measures**
Urine volume: at least 2.0 to 2.5 L/24 h
Urine density: <1.010 g/cm^3^
Fluid intake evenly distributed throughout the day
Fluid intake before going to bed
Replacement of extrarenal fluid losses caused by extensive physical activity, hot and/or dry environments, occupation, mental stress, and diarrhea
**Neutral Beverages**
Fruit tea, herbal tea, kidney tea, bladder tea
Tap water (attention must be paid to the sterility of water)
Mineral water with a low content of calcium, bicarbonate, and sulfate
**Alkalizing Beverages**
Mineral water with high bicarbonate (≥1500 mg/L) and low calcium content (<150 mg/L)
Orange juice
**Unsuitable Beverages**
Green tea, black tea, caffeinated coffee (maximum 0.5 L/day)
Sugar-sweetened soft drinks, including cola
Alcoholic beverages, including wine and beer

**Table 2:** Oxalate content of beverages.

Beverage	Description	Oxalate Content	References
		(mg/100 mL)	
**Vegetable Juices**			
Rhubarb nectar	60% juice	198	[56]
Beetroot juice	100% juice	60–70	[56]
Tomato juice	100% juice	4.1–8.1	[55,56]
Multi-vegetable juice	100% juice	3.6–8.5	[56]
Carrot juice	100% juice	4.6–5.8	[55,56]
Soybean drink	62% soymilk	4.4	[56]
**Fruit Juices**			
Grape juice, red	100% juice	2.1–3.9	[55,56]
Grape juice, white	100% juice	1.5	[55,56]
Apple juice	100% juice	0.9	[55,56]
Grapefruit juice	100% juice	0.1–0.3	[55,56]
Orange juice	100% juice	<d.l.–0.2	[55,56]
**Tea and Coffee**			
Green tea	Brewed	0.8–14.0	[59,60]
Black tea	Brewed	3.9–6.3	[60]
Iced tea	Ready-to-drink	0.3–2.0	[60]
Coffee	Filtered	0.6	[55]
**Beer and Wine**			
Malt beer		1.8	[60]
Wheat beer		1.3–1.8	[55,60]
Pils		1.3	[60]
Red wine		0.7–1.3	[60]
White wine		0.3	[60]

d.l., detection limit.

**Table 3:** Oxalate-rich foods.

Food	Description	Oxalate Content	References
		(mg/100 g)	
**Vegetables**			
Spinach	Raw	1959	[143]
Sorrel	Raw	1391	[143]
Rhubarb	Raw	1235	[143]
Mangold	Raw	874	[143]
Sweet potato	Raw	496	[145]
Okra	Raw	317	[145]
Beetroot	Raw	160	[143]
Olive, green	Canned	45.7	[55]
**Legumes**			
Beans, white	Seeds, dry	548	[145]
Soybeans	Seeds, dry	277	[145]
Quail beans	Seeds, dry	177	[145]
Kidney beans	Seeds, dry	74.6	[145]
Green beans	Raw	65.2	[145]
**Fruits**			
Star fruit	Raw	295	[55]
Elderberry, black	Raw	72.1	[55]
Blackberry	Raw	29.2	[55]
Gooseberry, green	Raw	27.0	[55]
Kiwi fruit	Raw	23.0	[55]
Fig	Raw	20.5	[55]
**Pseudocereals**			
Amaranth	Nuts	232	[143]
Quinoa	Nuts	184	[143]
Buckwheat	Nuts	143	[143]
**Cereals**			
Wheat	Bran	457	[144]
Wheat	Wholegrain flour	70.0	[144]
Bulgur		59.4	[144]
Couscous		65.2	[144]
**Nuts and Cocoa**			
Sesame		3800	[55]
Almond		383	[55]
Hazel nut		167	[55]
Pistachio		56.5	[55]
Cocoa powder		567–619	[55,145]
**Herbs and Spices**			
Licorice	Root	3569	[145]
Blue fenugreek	Powder	1246	[145]
Pepper, black	Grinded	623	[55]
Parsley	Raw	136	[55]

**Table 4:** Dietary recommendations for calcium oxalate stone patients.

Urinary Risk Factor	Limit	Recommendation
Urine volume	Urine volume < 2.0 L/24 h	Fluid intake that achieves urine volume ≥ 2.0 to 2.5 L/24 h
		Neutral and alkalizing beverages
Hypercalciuria	Calcium > 5 mmol/24 h	Calcium intake: 1000 to 1200 mg/day
		Protein intake: 0.8 to 1.0 g/kg normal body weight/day
		Sodium chloride intake: <6 g/day
		Increased intake of vegetables and fruits
Hyperoxaluria	Oxalate > 0.5 mmol/24 h	Low dietary oxalate intake
		Calcium intake: 1000 to 1200 mg/day (IH)
		Calcium supplementation (EH)
Hyperuricosuria	Uric acid > 4 mmol/24 h	Protein intake: 0.8 to 1.0 g/kg normal body weight/day
		Reduced dietary purine intake
		Increased intake of vegetables and fruits
Hypocitraturia	Citrate < 1.7 mmol/24 h	Protein intake: 0.8 to 1.0 g/kg normal body weight/day
		Increased intake of vegetables and fruits

IH, idiopathic hyperoxaluria; EH, enteric hyperoxaluria.

## Data Availability

Not applicable.
